# Examining multimorbidity differences across racial groups: a network analysis of electronic medical records

**DOI:** 10.1038/s41598-020-70470-8

**Published:** 2020-08-11

**Authors:** Pankush Kalgotra, Ramesh Sharda, Julie M. Croff

**Affiliations:** 1 Raymond J. Harbert College of Business, Auburn University, Auburn, AL USA; 2 Spears School of Business, Oklahoma State University, Stillwater, OK USA; 3 Center for Health Sciences, National Center for Wellness and Recovery, Oklahoma State University, Tulsa, USA

**Keywords:** Public health, Comorbidities

## Abstract

Health disparities across ethnic or racial groups are typically examined through single behavior at a time. The syndemics and multimorbidity health disparities have not been well examined by race. In this study, we study health disparities by identifying the networks of multimorbidities among individuals from seven population groups based on race, including White, African American, Asian, Hispanic, Native American, Bi- or Multi-racial and Pacific Islander. We examined a large electronic medical record (EMR) containing health records of more than 18.7 million patients and created multimorbidity networks considering their lifetime history from medical records in order to compare the network properties among seven population groups. In addition, the networks at organ system level depicting the relationship among disorders belonging to different organ systems are also compared. Our macro analysis at the organ-level indicates that African-Americans have a stronger multimorbidity network followed by Whites and Native Americans. The networks of Asians and Hispanics are sparse. Specifically, the relationship of infectious and parasitic disorders with respiratory, circulatory and genitourinary system disorders is stronger among African Americans than others. On the other hand, the relationship of mental disorders with respiratory, musculoskeletal system and connective tissue disorders is more prevalent in Whites. Similar other disparities are discussed. Recognition and explanation of such differences in multimorbidities inform the public health policies, and can inform clinical decisions as well. Our multimorbidity network analysis identifies specific differences in diagnoses among different population groups, and presents questions for biological, behavioral, clinical, social science, and policy research.

## Introduction

The health disparities across ethnic or racial groups can be attributed to the social, economic, environmental or geographic disadvantage to some groups in addition to the characteristics historically linked to discrimination or exclusion^1^. Elimination of racial disparities is a critical goal of public health systems. The Healthy People is an initiative that includes 10-year objectives for the health of the nation. The goals of Healthy People continue to emphasize a commitment on the elimination of health disparities: Healthy People 2000 and Healthy People 2010 goals included an overall reduction in health disparities; and for Healthy People 2020, the goal was to create systems of health equity^2^. Moreover, the American Public Health Association continues to advocate for health equity through conference themes.

In the United States, race and ethnicity are social and cultural constructs^3^, with minor support for biological and genetic determinants of health. Identifying racial and ethnic health disparities, whether social, cultural, or biological, are necessary to identify the underlying causes, and then to create interventions to reduce or eliminate such disparities^4^. In this study, we focus on identifying the multimorbidity (a medical condition when two or more diseases are diagnosed in a patient simultaneously) differences by race/ethnicity using the medical data of more than 18.7 million patients in US hospitals, and propose some research questions.

Administrative data from health systems have been used in the past to identify health disparities. However, not much work has been done to identify health disparities from comorbidity perspective, where in the presence of a primary diagnosis patients are diagnosed with an additional disease^5^. The presence of multiple diseases indicates a different health status of a patient as opposed to any single disease. The consequence of the presence of multiple diseases on a patient’s health is different from their individual independent effects as postulated by Singer in the description of syndemics theory^6^. In other words, the whole is greater than the sum of its parts. It is important to understand the health disparities from comorbidity and multimorbidity lens because if patients belonging to a particular group develop distinct diseases simultaneously, it can have a significant impact on clinical and policy decisions. Therefore, finding racial health disparities from multimorbidity lens will generate additional insights. Our paper is a descriptive analytics paper where our main contribution is to uncover interesting comorbidity patterns across ethnic/racial groups based upon a very large data warehouse, and pose new questions for future research.

Comorbidity/multimorbidity research has largely focused on the categorization of patients and comorbidities, with race/ethnicity being the secondary focus as summarized in Supplementary Appendix A. For example, Lankarani and Assari^7^ selected diabetic patients and analyzed the comorbidity differences across Non-Hispanic Whites, African Americans, and Caribbean Blacks. Similarly, Lee and authors^8^ analyzed survey responses of COPD patients and identified comorbidities in Whites, Blacks, Hispanics and Koreans. However, much of the previous research on comorbidities has inadequately examined the role of race, because of the inclusion of only some groups. Except for Erving in 2017^9^ most studies have only considered whites and blacks with a few adding Hispanics. However, the sample size in study by Erving^9^ was quite small (i.e. 12,787 participants) relative to the current study.

Because health disparities with respect to the comorbidities across race are prevalent, it is important to find such comorbidity or multimorbidity differences across all the groups. These types of analyses are only possible if a large dataset representing a diverse population is available. To address this gap, we analyze an Electronic Medical Record (EMR) containing health history of 18.7 million patients and compare multimorbidities/comorbidities across seven groups: White, African-American, Asian, Hispanic, Native American, Pacific Islander and Biracial. In addition, to find all types of multimorbidities across population groups hidden in a large EMR, an advanced data analytics method, network analysis, is applied. We use network analysis to model all comorbidities together through a network of diagnoses^10^. Additionally, our analytical model allows a specific focus on organ system level multimorbidities, in order to create networks at organ system level to explore the relationship between diagnoses belonging to different organ systems. Glicksberg and authors^11^ applied a network method to find health disparities across Whites, African Americans and Hispanics and found 51 hub diseases in the network. However, to the best of our knowledge, no one has presented the network approach to find comorbidity and multimorbidity differences at organ system level among seven population groups with a large EMR dataset.

We contribute by presenting a novel method to study racial/ethnic health disparities at the multimorbidity level, which can be explained through the syndemic theory. Notably, the syndemic theory helps us generate new research questions for future research by considering synergistic interaction of social, cultural, psychological, and biological factors^12,13^. In the past, syndemic theory has been applied to study a particular situation (e.g. depression-diabetes by Mendenhall^13^; stress, diabetes, and infection by Mendenhall and authors^14^) where the focus was not on the method. In this study, we contribute by presenting a network method to identify multiple combination of diseases across different groups. Notably, we acknowledge that the differences are not ONLY about races being sicker in specific ways, it is also about diagnoses and treatment of specific groups of people. Our study does not distinguish between the biological, social, political and other external factors.

## Method

We obtained data from the Center for Health Systems Innovation at Oklahoma State University, which conducts research on HIPAA compliant patient data provided by Cerner Corporation, a major Electronic Medical Record (EMR) provider. The data warehouse contains sixteen years of health records on the visits of 24.7 million unique patients across 662 US hospitals (2000—2015) marked with at least one diagnosis or symptoms. Following the CRoss-Industry Standard Process for Data Mining (CRISP-DM)^15,16^, we assessed the quality of data, cleaned it and then prepared it for analysis. For our analysis, we disregarded hospital visits where patients were not diagnosed with a disease or diagnosed with just a general symptom. This left us with 22.1 million patients. The race information in the EMR has multiple discrepancies as recently discussed by Polubriaginof and authors^17^ and Sholle and authors^18^. We used multiple approaches to resolve data related issues. For instance, the patients marked with different races across hospital visits were considered erroneous and removed for further analysis. In addition, the patient records without any race information were also dropped. After data cleaning and filtering, we extracted medical records (18.7 million patients) for different races in seven different datasets from the pseudo-population dataset for comparing multimorbidities/comorbidities. Finally, we had 14.1 million Whites, 3.46 million African Americans, 592,725 Hispanics, 400,521 Asians, 157,880 Native Americans, 25,414 Pacific Islanders and 29,654 Biracial patients’ information recorded by the hospitals or medical providers. Clearly, there is a large difference among sample sizes of different groups. We have addressed the issue of comparing unequal sample sizes in the following section.

The summary statistics of the dataset is included in Table 1. This dataset represents medical records across a large swath of the US and covers hospital visits between 2000 and 2015. Of course, not all the hospitals adopted the EMR at the same time, so it does reflect different time periods at different hospitals. However, there is no known pattern for adoption of the EMR based upon races, so the results presented here are robust. The average number of visits within the dataset is the highest for African Americans (4.83), followed by Whites (4.69), Native Americans (3.57), Asians (3.13) and Hispanics (2.6). The same pattern has been observed in the average number of distinct diagnoses per patient in different categories. The average age of a patient at the time of a hospital visit shows that Hispanics have the lowest average age followed by Native Americans, African Americans, Asians and Whites. We also calculated the proportion of patients enrolled in Medicare and Medicaid plans. The regional disparity, number of visits in different types of hospitals and proportion of inpatients and emergency visits are also included. These numbers reflect a typical US demographic distribution across the US among regions, urban vs rural area, etc., and lend credence to the comorbidity results obtained in this study.

**Table 1:** Summary statistics.

	African American	White	Hispanic	Asian	Native American
Average no. of visits per patient	4.83	4.694	2.571	3.129	3.572
Average number of distinct diagnoses per patient	5.86	5.71	3.32	3.78	4.81
Average age per visit	39.93	50.45	28.4	43.7	38.55
No. of patients with Medicare	16.69%	26.33%	6.55%	10.97%	15.67%
No. of patients with Medicaid	17.48%	9.6%	27.8%	9.3%	30.58%
No. of visits from census regions (Northeast, Midwest, South and West respectively)	16.7%,29.9%, 51.7%,1.5%	40.1%,26.9%, 22.0%,10.9%	35.3%,14.7%, 32.7%,17.3%	28.3%,28.5%, 12.0%,31.2%	10.3%,21%, 22.0%,46.7%
No. of visits in different hospitals (bed-size < 5, 6–99, 100–199, 200–299, 300–499 and 500 + respectively)	8%2.4%,6.4%, 27.9%,23%, 32.3%	10.3%,11.8%,13.1%,21.1%,21.9%,21.8%	3%,5.6%,6%, 39.3%,35.2%,10.9%	17.9%,3.9%,5.3%, 20%,32.4%, 20.4%	16.3%,17.8%, 9%,12.7%, 19.5%,24.7%
No. of inpatient visits and emergency visits respectively	9.6%, 29.9%	9.1%, 16%	10.3%, 38.8%	10.3%, 14.4%	8.3%, 29.3%

### Measuring multimorbidity and network

We measure multimorbidity as the presence of multiple diseases in the lifetime history of a patient^19^. Traditional definition of a multimorbidity only considers the diseases present in a patient at the time a patient is examined or during the specific encounter, without considering the past medical history. Several researchers have suggested to use the past medical history for understanding the current situation of a patient^20,21^. Similarly, for our purpose, we measure the lifetime associated multimorbidity of a patient. This historical and current multimorbidity gives a better trajectory of a patient's diagnosis and potential treatment. It is analogous to a physician asking a patient for the past medical history during a visit. In addition, our measurement controls for the presence of some chronic diseases, which might exist multiple times across hospital visits in an EMR. As per our measurement, the EMR recording of a disease over multiple hospitals visits is only considered once. However, there is a concern of false positives with the association between diagnoses occurring after a long period of time. This concern is eased because of the short duration of the database (16 years) and statistical analysis performed on millions of patients.

Using the health history from the administrative data, we developed a multimorbidity network for each group containing a set of nodes connected through edges. In our network, nodes represented diagnoses classified according to the International Classification of Diseases, Ninth Revision, Clinical Modification (ICD-9-CM). An ICD-9 code of a diagnosis has three, four or five digits (xxx.xx). The first three digits represent the broader category of a disease whereas the fourth and fifth digits represent the sub-divisions of a disease. We aggregated ICD-9 codes to three-digit level to consider variations of the same disease as one node in the network.

An edge or a connection between two diagnoses was created based on their co-presences in patients of all age groups*.* An edge was without a direction as our focus is not to establish causality of a comorbidity. Mathematically, the strength of an edge was calculated using a cosine measure known as Salton Cosine Index^22^. In the past, a connection between diagnoses or comorbidities have been modeled using a Pearson’s Correlation Coefficient for binary variables^23,24^. However, it depends on the sample size. For our comparative analysis, we cannot use correlation to create comorbidity networks as the sample size differences among seven population groups are huge. In contrast, we adapt Salton Cosine Index (SCI) for binary variables that does not depend on the total number of patients^25^. It measures the prevalence of a relationship between two diagnoses considering their individual prevalence. SCI has been used in epidemiology to study comorbidity^19^, and is thus an acceptable and preferred measure.

Salton Cosine Index, *SCI*, for a pair of diagnoses *i* and *j* is calculated as in Eq. (1), where *c*_*ij*_ is the number of co-occurrences of diagnoses *i* and *j, c*_*i*_ is the prevalence of diagnosis *i* and *c*_*j*_ is the prevalence of diagnosis *j*.

1 $$SCI_{ij} = \frac{{\left( {c_{ij} } \right)}}{{\sqrt {\left( {c_{i} *c_{j} } \right)} }}$$

Following Kalgotra, Sharda and Croff study^19^, the statistical significance of SCI of each pair was determined by assessing the relationship between correlation and SCI. We selected a cut-off for SCI as 0.04 in the entire dataset at which the number of comorbidities was equal to the number of significantly correlated comorbidities at 1% significance level. Using the significance level of 1%, we mitigate the concern of false positives i.e. the concern of selecting connections with low correlations. We used the SCI cut-off of 0.04 for creating different networks for seven groups of patients. To get rid of the disease connections occurring by chance, the disease-pairs occurring less than the average are removed in each group.

Although SCI is robust to the sample size, we decided to perform comparative (matched) analysis of population groups using both equal and unequal sample of patients. Originally, we had 14.09 million Whites, 3.46 Million African Americans, 592,725 Hispanics, 400,521 Asians, 157,880 Native Americans, 25,414 Pacific Islanders and 29,654 Biracials. The sample size of latter two groups is relatively small and therefore, we study them separately in Supplementary material online. For the remaining five groups, we extracted number of patients (random sample) equal to the smaller group i.e. 157,880 to perform analysis of equal samples. This led us compare the multimorbidity networks at the same level with balanced samples.

Seven different multimorbidity networks including two in Supplementary material were created and compared using centrality measures such as degree and weighted degree centrality^26^. In a multimorbidity network, the degree centrality of a disease (node) denotes the number of direct connections with other diseases. The weighted degree centrality of a disease considers the strength of relationships with others and is calculated as a weighted sum of the strengths of relationships. Finally, to perform a macro level analysis, the connections are aggregated to the organ system level defined by the ICD-9-CM codebook.

## Results and Discussion

Table 2 lists the properties of multimorbidity networks of all population groups considered in this study. To validate the robustness of SCI with different sample sizes, we created networks using the entire population of a group in our EMR and its corresponding equal random sample. Table 2 lists the number of connections in the both types of networks. The proportion test (chi-squared (χ^2^) test) comparing the number of connections present with respect to the maximum number of possible connections found that there is no statistical difference between the two types of networks (African American: χ^2^ = 0, *p* = 1; White: χ^2^ = 0.001, *p* = 0.98; Hispanics: χ^2^ = 0.059, *p* = 80; Asian: χ^2^ = 0.033, *p* = 0.85). Because the networks with small sample preserved the properties of population networks, we used networks created using equal (small) samples for comparative analysis.

**Table 2:** Race comorbidity networks properties.

Race/ethnicity	Population/unequal sample			Equal sample (157,880 patients)			
	Number of patients	Number of diagnoses	Comorbidities	Number of diagnoses/ nodes	Comorbidities/ connections	Average degree^a^	Average weighted degree^b^
African American	3,461,945	908	16,596	891	15,863	35.6	2.9
White	14,093,395	908	14,177	884	13,377	30.3	2.4
Hispanic	592,725	898	6,467	871	6,361	14.6	1.16
Asian	400,521	900	8,600	889	8,628	19.4	1.5
Native American	157,880	887	11,560	887	11,560	26	2.18

^a^Average degree of at least one race is different from other (F value = 28.44, *p* < 0.0001), ^b^Average weighted degree of at least one race is different from other (F value = 27.20, *p* < 0.0001).

**Figure 1:**
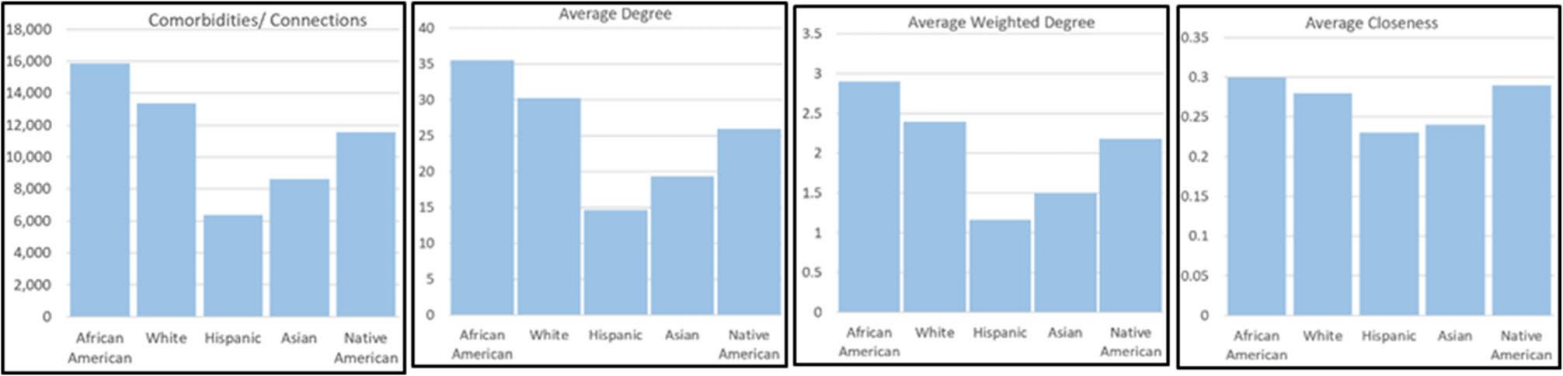
Race comorbidity networks properties.

Table 2 lists the features of networks of five population groups. Figure 1 visually presents the network properties. All networks have an almost equal number of diagnoses. The number of diagnoses pairs i.e. connections in African-Americans were the highest followed by the White, Native Americans, Asians and Hispanics. Clearly, there are large significant differences in these numbers ranging from 15,863 to 6,361 (Proportion test, χ^2^ = 6.9*10^8^, *p* < 0.0001). The network corroborates the health disparities in term of relationship of a disease with other diseases. The average degree and weighted degree centralities of African-American network are the highest indicating the patients often get diagnosed with multiple diseases. On the other extreme, Hispanics and Asians have the lowest numbers. To confirm the robustness of our findings, we have conducted several validity-checks to assess the effect of other confounding factors. The results of validity-checks are provided in Supplementary Appendix B where we performed matched-analyses accounting for the number of hospital visits, age, sample size and time duration of the database. The results are consistent and thus, confirm the robustness of our findings.

**Figure 2:**
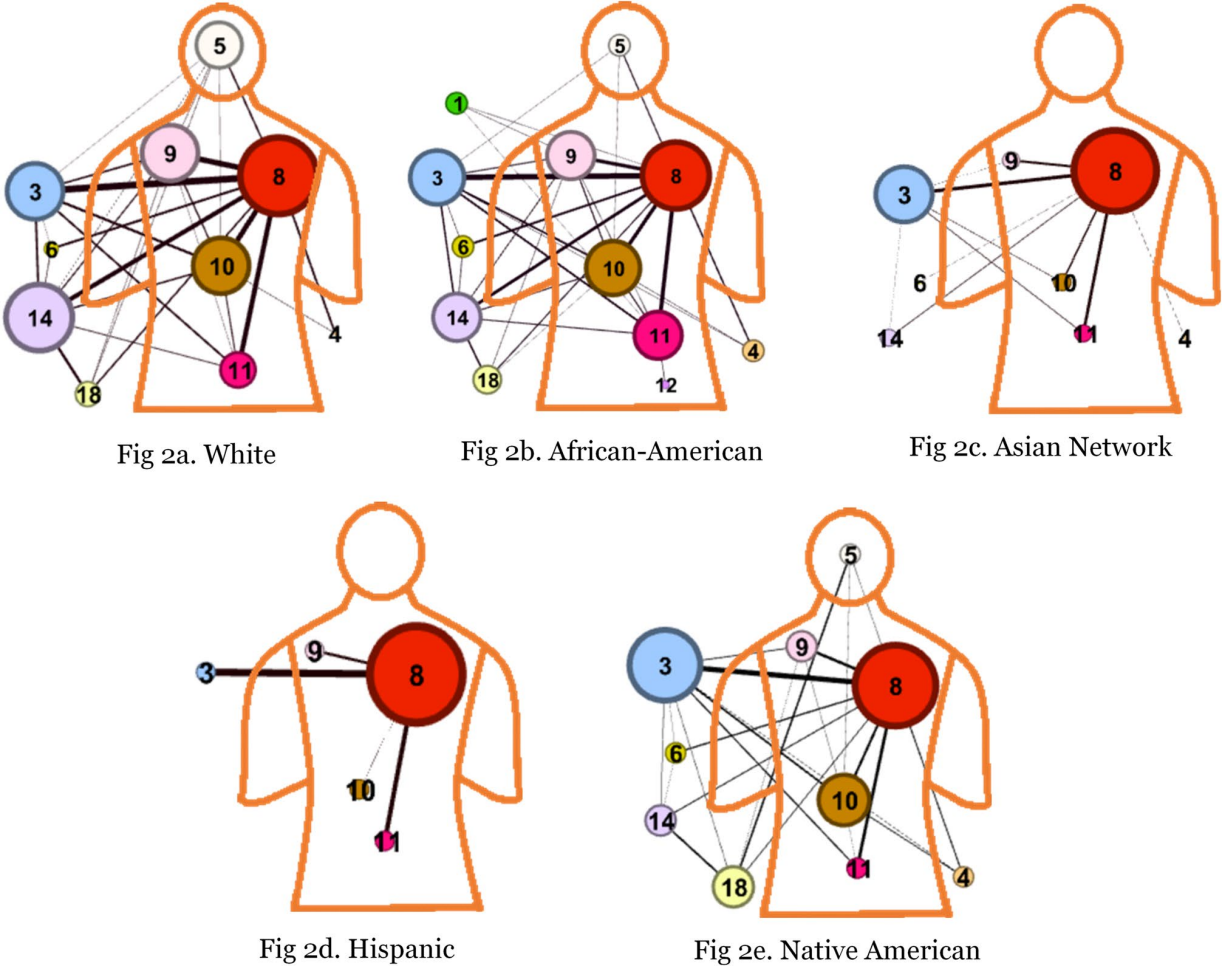
Multimorbidity network by race at organ system level.

The multimorbidity disparities are more apparent at the organ-system level. Figure 2a through e present the visualizations where an edge between two organ systems was created by taking the aggregated sum of SCI of pairs belonging to two organ systems. We highlight the connections between diagnoses of different organ systems if their aggregated weight is more than ten. This cut-off of ten is to study the most prevalent relationships. However, one could select a lower cut-off to analyze the rare connections. Notably, we have identified the comorbidities among diseases across the patients and then aggregated to the organ system level. We have used ICD-9-CM codebook for defining an organ system and classified the diseases into eighteen categories. This is different from the approach where diseases are first aggregated at the organ system level and then find relationship between organ systems. Following this approach would lose the comorbidities at the disease level. However, our approach is better than the later because our method preserves the granular comorbidities at the disease level.

In the organ-level visualizations (Fig. 2a–e), a node represents the collection of disorders belonging to an organ system. We mapped the organ-system level nodes at their location on the outline of a human body. In the ICD-9 classification (see Supplementary Appendix C), some organ systems are present on a specific location in human body such as circulatory system (class-8), mental disorders (class-5), digestive system (class-10), respiratory system (class-9), and genitourinary system (class-11). In Fig. 1a–e, these organ-level nodes are marked on their respective location on the human body outline. However, the classes that cannot be mapped on a particular location on human body are sketched outside of the human body outline such as infectious and parasitic diseases (1), neoplasms (2), endocrine, nutritional and immunity disorders (3), blood and blood-forming organs disorders (4), nervous system disorders (6), sense organ disorders (7), pregnancy related diseases (12), skin diseases (13), musculoskeletal system disorders (14), congenital anomalies (15), perinatal period disorders (16) and injury/poisoning (18). In these visuals, the size of a node represents the number of connections to other nodes whereas the thickness of a connection between two nodes represents the aggregated weight i.e. tie strength. The same connections can be observed in Table 3 where a comparison is made among five networks.

**Table 3:**
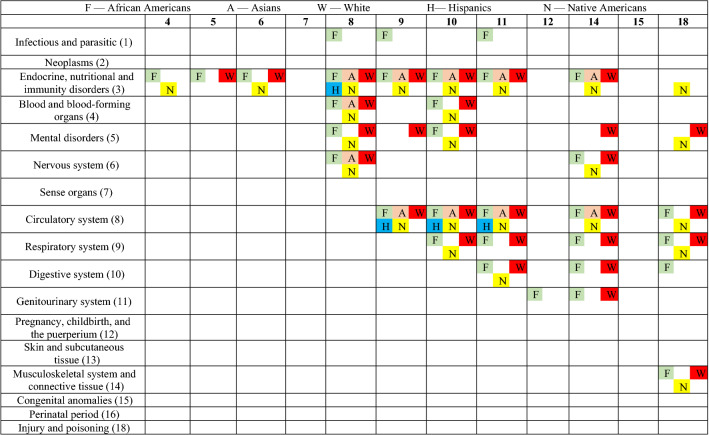
Presence/absence of organ system level comorbidities across races.

*Class 17 is general symptoms, and thus not included in the comorbidity analysis.

There are notable differences in the networks by race/ethnicity, with recent Hispanic and Asian populations having fewer connections in the network. The African American network (32 connections), the White network (29 connections), and the Native American network (24 connections) have similarly oriented and relatively dense networks of multi-morbidities. On the other hand, the Asian network (11 connections), and the Hispanic network (4 connections) networks are less dense. This raises a future research question. Could these differences be explained by healthy immigrant paradox^27^? Healthy Immigrant Paradox states that only the healthiest individuals are able to migrate to a new country, and therefore, remain healthier than individuals in the new country and healthier than individuals in their country of origin. The exception seems to be moving to the United States, where people (historically) had increased access to processed foods and sedentary lifestyles^28^. In addition, migrant groups have stronger social ties^29^. This argument is supported by literature but requires further enquiry.

Since the networks of migrant groups are very sparse, there are no specific comorbidity in their networks. However, some comorbidities are unique to other groups. African Americans have several comorbidities that are unique to this particular group. For example, the relationship of infectious and parasitic disorders with respiratory, circulatory and genitourinary system disorders is unique to the African American network. Could this relationship be attributed to the higher HIV cases in this group as indicated by the CDC report?^30^. In addition, our analysis raises several specific questions about the relationships of infectious and parasitic disorders with all other systems, which need more explanation. The relationship between digestive disorders, and injury and poisoning are stronger in the African American network than in other networks. Finally, the comorbidity network involving disorders of genitourinary system and pregnancy, childbirth, and the puerperium are more common in African Americans than others: a network supported by the known disparities in pregnancy-related outcomes among African American women^31^. In contrast, the relationship of mental disorders with respiratory, musculoskeletal system and connective tissue disorders is more prevalent in Whites. The comorbidities involving mental disorders and injury and poisoning is more dominant in the White and Native American networks. On the other hand, the comorbidity involving endocrine, nutritional and immunity disorders and injury and poisoning is stronger in Native Americans than others; notably the existing literature supports health disparities among Native Americans wherein unintentional injury occurs at 2.4 times the rate in Native Americans than Whites^32^ and higher risk of diabetes and comorbidities in this group^33^.

Comorbidities that are unique to a particular ethnic group open avenues for further research to develop explanations from biological, social, cultural and political perspective, which is at the heart of syndemics theory. It is important to understand how socioeconomic factors such as subjective relative wealth contribute to multimorbidity health disparities as argued by Smith and group^34^. In addition, it is important to investigate how the preventative care utilization across the ethnic groups (which may be affected by the demographic characteristics and social relationships) impacts the identified multimorbidity differences^35^. Likewise, could there be an impact of the differences in health insurance held by different population groups^36^, which may impact medical providers’ desire to provide just some basic care and release a patient as opposed to keeping them in the hospital longer and diagnose other comorbidities? Some studies hint of such differences. For example, see Spencer et al.^37^ and Lin et al.^38^. Notably, because individuals without insurance may be less likely to receive diagnoses for their illnesses until there was an event that resulted in hospitalization, and in that context, the focus is typically on a single diagnosis. However, among those who live in poverty and have access to government supplied health insurance, there is a higher use of medical care, and an increased likelihood of diagnosis and likely a lower level of resolving the chronic disease because of the syndemic effects on disease outside of the medicine received for the management of the chronic disease. Furthermore, it is crucial to examine how much the ethnic inequities in the personal health records (PHR) use and digital divide contribute to the comorbidity differences^39–41^. Moreover, social stigma has also been related to the health disparities in the past, and therefore this analysis supports the need for targeted interventions to address health related social stigma^42^. These finding also support the use of health literacy interventions in order to increase knowledge associated with specific comorbidity health disparities^43^.

## Conclusions

We established comorbidity differences across different US population groups based on race and ethnicity. Our multimorbidity network analysis of organ system level revealed differential comorbidity networks. We believe that our paper validates some past findings using a very large data warehouse, uncovers additional differences while illustrating new methods of presentation, and raises several research questions for clinical and policy research. As pointed out earlier, the disparities discussed are not ONLY due to the sickness differences among races, it is also about diagnoses and treatment of specific groups of people. Some factors are assessed through validity-checks in Supplementary Appendix B, but other biological, social and political factors can also be responsible for the disparities.

Medical treatment studies remain dominated by white males^44^, which may adversely impact the treatment of multimorbidity in different population groups. This needs to be addressed in future research. Our examination of the multimorbidity differences across races and ethnic groups strengthens the need for understanding the reasons for such disparities, and developing interventions to eliminate disparities. Such research is clearly interdisciplinary.

There are a few limitations of this study. First, although we analyzed millions of patient records to identify these differences across races, these are based on one specific EMR in US hospitals. Therefore, the history of patients recorded in other systems was not present in our analysis. By performing several validity-checks, we mitigated this issue. Also, the races are spread across the world^45^ but only US information was used in this paper. In future, we plan to use health records of patients outside the US. Second, we explained the multimorbidity differences among five population groups in the main text. There are multiple other groups which we could not include in our analysis due to small sample size. However, we have included the analysis of Pacific Islanders and Biracial in Supplementary Appendix D where small but sufficient number of patient records were available. Third, the characteristics of the population groups such as age were not the same in our dataset. However, the validation checks confirmed the robustness of our findings. Finally, our results included thousands of multimorbidities which could not be reported in the main text due to the information overload. Instead, we have attached supplementary materials containing information on relationship of every diagnosis with others. One can focus on one particular diagnosis and find multimorbidities in our provided material at https://bit.ly/2H4jMqT. Notably, the comorbidities presented in this paper were derived from our database using our method where the lifetime history of a patient was considered. A different definition of a multimorbidity can impact the results. The racial/ethnic comorbidity differences that have been presented in this study help focus on specific research opportunities for identifying gene-associations related to these specific comorbidities across different population groups.

Our data-driven study analyzed information from millions of patients that raises multiple questions. There are several variations in different population groups with respect to the multimorbidities that should be considered in treatment decisions and policy making. The large dataset such as ours can be used for public health surveillance. Our study opens up an exciting and important area of research for policy makers, economists, social scientists and medical experts to treat different groups of population differently by considering the combination of diseases.

## Supplementary information

Supplementary information.

## Data Availability

This work was conducted with data from the Cerner Corporation’s Health Facts database of electronic medical records provided by the Oklahoma State University Center for Health Systems Innovation (CHSI). We obtained permissions to use this database from CHSI. The dataset was released after the signature of a contract and can only be used for the current study, and so they are not publicly available. However, we provide multimorbidities data to conduct future research at https://bit.ly/2H4jMqT.
