# Supplementary material for: Examining multimorbidity differences across racial groups: a network analysis of electronic medical records
Source: Sci Rep. 2020 Aug 11;10:13538. doi: 10.1038/s41598-020-70470-8 (PMC7419498; doi:10.1038/s41598-020-70470-8)
Supplement: Supplementary file 1 — Supplementary information. [file 41598_2020_70470_MOESM1_ESM.pdf]

Examining Multimorbidity Differences across Racial Groups: A Network Analysis of Electronic Medical Records

Pankush Kalgotra, PhD
Assistant Professor
Raymond J. Harbert College of Business
Auburn University
Auburn, AL, US, 36849
pzk0031@auburn.edu

Ramesh Sharda, PhD
Vice Dean, Watson Graduate School of Management
Regents Prof. and Watson/ConocoPhillips Chair of Mgmt. Sc. & Info Systems
Spears School of Business
Oklahoma State University
Email: ramesh.sharda@okstate.edu

Julie M. Croff, PhD
Center for Health Sciences
Center for Wellness and Recovery
Oklahoma State University
julie.croff@okstate.edu

Appendix A. A list of papers studying comorbidity differences across races

Paper	Data	Race considered	Comorbidities observed	Comment
Lankarani & Assari [1]	603 diabetes patients from the National Survey of American Life (2001-2003)	Non-Hispanic Whites, African Americans, and Caribbean Blacks	Major depressive disorder, Arthritis/rheumatism, peptic ulcers, cancer, hypertension, chronic liver disease, chronic kidney disease, stroke, asthma, other chronic lung diseases, atherosclerosis, sickle cell disease, heart disease and glaucoma	Number of medical comorbidities was positively associated with lifetime MDD among non-Hispanic Whites and African Americans but not Caribbean Blacks with diabetes.
Lee et al. [2]	5,303 participants with COPD obtained from the U.S. National Health and Nutrition Examination Survey (NHANES) (2007–2012) and from the Korea NHANES (2007–2015)	Non-Hispanic black and white, Hispanics and Koreans with 40 to 79 years of age	Asthma, Hypertension, Dyslipidemia, Stroke, Myocardial infarction, Diabetes mellitus, Anemia, Osteoarthritis, Rheumatoid arthritis and Osteoporosis	Whites had the highest prevalence of dyslipidemia, myocardial infarction, osteoarthritis, and osteoporosis, while non-Hispanic Blacks had the highest presence of asthma, hypertension, stroke, diabetes mellitus, anemia and rheumatoid arthritis.
Watkins, Assari, & Johnson-Lawrence [3]	6082 respondents from National Survey of American Life (2001-2003)	Non-Hispanic Whites, African Americans, and Caribbean Blacks	Major depressive disorder, general anxiety disorder, Arthritis/rheumatism, peptic ulcers, cancer, hypertension, chronic liver disease, chronic kidney disease, stroke, asthma, other chronic lung diseases, atherosclerosis, sickle cell disease, heart disease and glaucoma	Lifetime major depressive disorder was associated with at least one chronic medical condition among African Americans and Caribbean Blacks but not non-Hispanic Whites.
Sanchez et al. [4]	290 participants from nine community-based substance abuse treatment programs across the United States	Non-Hispanic Black, non-Hispanic White and Hispanic	Stimulant use disorders, mental and physical health disorder	Black participants reported more comorbid medical conditions, poorer health status, and lower physical and cognitive functioning than other groups.
Williams et al. [5]	1,314 patients in the Veterans Health Administration diagnosed with early-stage non–small-cell lung cancer in 2007	White and black	Lung cancer related comorbidities of psychiatric, rheumatologic, cardiovascular, respiratory, gastrointestinal, renal, endocrine, neurologic, immunologic logic system disorders	Comorbidities more prevalent in blacks included hypertension, liver disease, renal disease, and illicit drug abuse. However, respiratory disease was more prevalent in whites.
Glicksberg et al. [6]	1,025,573 patients from one hospital	White, African American and Hispanic/Latino	All types of comorbidities observed in EMR	Identified 51 key hub diseases that are the focal points in the race-centric network
Erving [7]	12,787 participants from Collaborative Psychiatric Epidemiology Surveys (CPES)	Non-Latino Whites, African Americans, Caribbean Blacks, Spanish Caribbean Blacks, Mexicans, Cubans, Puerto Ricans, Other Latinos, Chinese, Filipinos, Vietnamese, and Other Asian Americans.	Physical and psychiatric health problems	Puerto Rican men have significantly higher risk of Physical and psychiatric comorbidity in comparison to Non-Latino White men. Among women, Blacks and Cubans were more likely than Non-Latino Whites to experience physical and psychiatric comorbidities
Opara et al. [8]	30,852 adults from the National Health Interview Survey, 2003	African Americans, Whites, and Hispanics	Comorbidities related to hypertension such as diabetes mellitus, depression, other endocrine disorders, circulatory disorders, and hypercholesterolemia	African Americans had the highest prevalence of hypertension-diabetes mellitus comorbidity as compared to White and Hispanics.
Tammemagi et al. [9]	906 patients with breast cancer between 1985 and 1990 from a health system	Black and White women	All types of comorbidities observed with breast cancer	More black patients die of comorbidities than of breast cancer.
Richardson et al. [10]	974 Veterans in care for HIV in 2013	Black and White	Hypertension, diabetes, lipids, acute phase depression, Hepatitis C, chronic kidney disease and substance use disorder	Black Veterans were more likely than white Veterans to be diagnosed with hypertension, substance abuse, Hepatitis C and chronic kidney disease. Black patients were less likely than whites to receive treatment to control viral, hypertension, diabetes and monitor lipids.
Our study	18.7 million patients from 662 US hospitals	White, African-American, Asian, Hispanic, Native American, Pacific Islander and Bi- or Multi-racial	All types of comorbidities observed in EMR	Identified comorbidity differences across seven races through organ system level networks.

Appendix B. Validity Checks

We performed several validity-checks to assess the robustness of our analysis and results. We accounted for the number of hospital visits, age, sample size and time duration of the database. Notably, the magnitude of comorbidities in these comparisons is smaller as compared to the numbers in main paper because the only subset of diseases are considered. To find out the impact of number of visits by a patient on the number of comorbidities, we performed analysis for patients with different races having exactly three visits in the database. We found the same pattern as reported earlier i.e. African-Americans have the highest number of comorbidities followed by Whites, Native Americans, whereas Asians and Hispanics have the least number. This shows that the smaller number of comorbidities in the immigrant groups is not a mere consequence of number of times they visit the hospital. Similarly, to study the impact of age on the comorbidity differences across races, we conducted a matched analysis by taking a subset of patients with exactly three visits and are within the age range of 31 and 50. Again, we found the same pattern with Hispanics and Asians having the least number of comorbidities. As discussed in the method section, we had created networks by taking an equal sample of patients in each race as reported in Table 2. However, to assess the validity of results, we performed the same analysis by taking another random sample of patients. The number of comorbidities in each race from another sample is consistent with the numbers in Table 2. Since the database we had access to is only 16 years, we performed a comparative analysis of patients with different time duration in the EMR. We considered the patients where the first and last visit of a patient in our database is within 365 days. Again, we found that Asians and Hispanics have the least number of comorbidities. However, within the shorter period, Native Americans had the highest number indicating longer period for other races present more comorbidities. This put forwards a future research question for researchers. Overall, the pattern among Whites, African Americans, Hispanics and Asians is consistent. All these validity checks confirm the robustness of our results. Below, we present all validity checks in detail.

1) To find the comorbidity differences across patients with different races having exactly the same number of visits.

   We included the number of comorbidities for patients with different races with exactly three visits in the database. Table B1 includes the number of comorbidities in a subset of patients with three visits in each race. The pattern here is the same as reported in the main paper i.e. African-Americans have the highest number of comorbidities followed by Whites, Native Americans, whereas Asians and Hispanics have the least number.

   In all validity checks, we followed the same method of finding comorbidities as specified in the paper by taking the Salton Cosine Index cut-off of 0.04 for a pair of diseases.

	**Number of patients**	**Comorbidities/ Connections**
**African American**	339,689	6,003
**White**	1,345,510	5,887
**Hispanic**	52,290	3,645
**Asian**	36,673	5,258
**Native American**	15,467	5,847

Table B1. Comorbidities in patients with three visits

2) To study the effect of age on the number of comorbidities across races.

We conducted the analysis by taking a subset of patients with three visits and are within the age range of 30 and 50. Again, we found the same pattern with Hispanics and Asians to be the least number of comorbidities as in Table B2 below.

	Number of patients	Comorbidities/ Connections
**African American**	98,796	4,329
**White**	395,796	4,104
**Hispanic**	14,291	2,661
**Asian**	12,764	3,104
**Native American**	4,849	4,098

Table B2. Comorbidities in Patients with three visits and age 31-50

3) To compare another sample of patients

We had taken multiple samples and reran the analysis. For example, given below are the results of another sample of EMR between races and the number of comorbidities. Our results (Table B3) are again consistent with the results reported in the main text.

	Number of visits	Comorbidities/ Connections
**African American**	157,222	16,031
**White**	158,038	13,702
**Hispanic**	157,807	6,211
**Asian**	158,104	8,562
**Native American**	94,818	11,367

Table B3. Comorbidities in another random sample

4) To study the patients with different time duration in the EMR.

To address this, we considered the patients where the first and last visit of a patient in our database is within 365 days (Table B4). Again, we found that Asians and Hispanics have the least number of comorbidities. However, within the shorter period, Native Americans had the highest number indicating longer period for other races present more comorbidities. This puts forward a future research question for researchers. Overall, the pattern among Whites, African Americans, Hispanics and Asians is consistent.

	Number of patients	Comorbidities/ Connections
**African American**	2,113,323	5,962
**White**	8,885,568	5,605
**Hispanic**	453,890	3,401
**Asian**	298,137	4,138
**Native American**	119,660	6,199

Table B4. Comorbidities in the patients with maximum duration of 365 days

Appendix C

Class 1 - 001–139: infectious and parasitic diseases
Class 2 - 140–239: neoplasms
Class 3 - 240–279: endocrine, nutritional and metabolic diseases, and immunity disorders
Class 4 - 280–289: diseases of the blood and blood-forming organs
Class 5 - 290–319: mental disorders
Class 6 - 320–359: diseases of the nervous system
Class 7 - 360–389: diseases of the sense organs
Class 8 - 390–459: diseases of the circulatory system
Class 9 - 460–519: diseases of the respiratory system
Class 10 - 520–579: diseases of the digestive system
Class 11 - 580–629: diseases of the genitourinary system
Class 12 - 630–679: complications of pregnancy, childbirth, and the puerperium
Class 13 - 680–709: diseases of the skin and subcutaneous tissue
Class 14 - 710–739: diseases of the musculoskeletal system and connective tissue
Class 15 - 740–759: congenital anomalies
Class 16 - 760–779: certain conditions originating in the perinatal period
Class 17 - 780–799: symptoms, signs, and ill-defined conditions
Class 18 - 800–999: injury and poisoning

Appendix D

The analysis of Pacific Islanders and Biracial was not included in the main text due to the small sample size. However, the use of Salton Cosine Index to develop networks mitigates the concern of sample size as it is less affected by the sample size. The properties of two networks are presented in Table D1. The density of Pacific Islanders network was 4.39% but the density of biracial network of diagnoses was only 1.7%. The node centralities such as average degree and weighted degree also tell the same story where these metrics are higher for Pacific Islanders. When the same networks were aggregated at the organ system level (See Fig. D1 and D2) and with the selected threshold of selecting the edges, we found only one connection between disorders of circulatory system and disorders of endocrine, nutritional and metabolic diseases, and immunity. On the other hand, the Pacific Islander network was very dense with 36 edges.

Pacific Islander	Biracial
**No. of patients**	
25,414	29,654
**Number of unique diagnoses**	
829	803
**Number of Connections**	
15,064	5,718
**Average Degree**	
36	14.2
**Average Weighted Degree**	
2.9	1.14

Table D1. Race Comorbidity Networks Properties

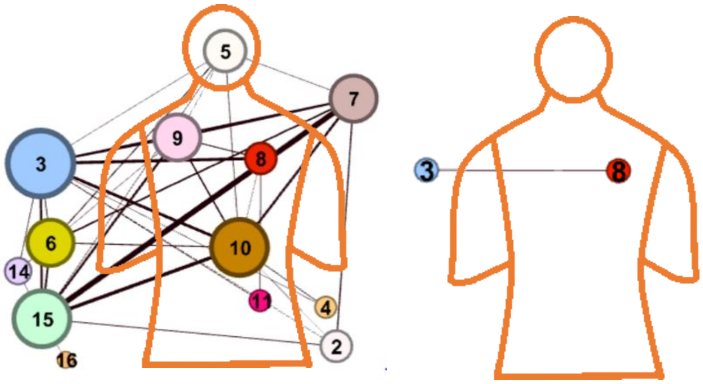

Figure D1. Pacific Islander   Figure D2. Biracial Network

Figure D. Pacific Islander Network and Biracial Networks

References

1 Lankarani MM, Assari S. Association between number of comorbid medical conditions and depression among individuals with diabetes; race and ethnic variations. *Journal of Diabetes & Metabolic Disorders.* **14,** 56 (2015).

2 Lee H, Shin SH, Gu S, et al. Racial differences in comorbidity profile among patients with chronic obstructive pulmonary disease. *BMC medicine.* **16,** 178 (2018).

3 Watkins DC, Assari S, Johnson-Lawrence V. Race and ethnic group differences in comorbid major depressive disorder, generalized anxiety disorder, and chronic medical conditions. *Journal of racial and ethnic health disparities.* **2,** 385-394 (2015).

4 Sanchez K, Chartier KG, Greer TL, et al. Comorbidities and race/ethnicity among adults with stimulant use disorders in residential treatment. *Journal of ethnicity in substance abuse.* **14,** 79-95 (2015).

5 Williams CD, Stechuchak KM, Zullig LL, et al. Influence of comorbidity on racial differences in receipt of surgery among US Veterans with early-stage non–small-cell lung cancer. *Journal of Clinical Oncology*. **31,** 475 (2013).

6 Glicksberg BS, Li L, Badgeley MA, et al. Comparative analyses of population-scale phenomic data in electronic medical records reveal race-specific disease networks. *Bioinformatics*. **32,** i101-110 (2016).

7 Erving CL. Physical-psychiatric comorbidity: patterns and explanations for ethnic group differences. *Ethnicity & health*. **23,** 583-610 (2018).

8 Opara F, Hawkins K, Sundaram A, et al. Impact of comorbidities on racial/ethnic disparities in hypertension in the United States. *ISRN Public Health.* (2013).

9 Tammemagi CM, Nerenz D, Neslund-Dudas C, et al. Comorbidity and survival disparities among black and white patients with breast cancer. *Jama*. **294,** 1765-1772 (2005).

10 Richardson KK, Bokhour B, McInnes DK, et al. Racial disparities in HIV care extend to common comorbidities: implications for implementation of interventions to reduce disparities in HIV care. *Journal of the National Medical Association*. **108,** 201-210 (2016).
